# The association of bullying and self-esteem with psychotic-like experiences and clinical high risk for psychosis symptoms

**DOI:** 10.1186/s13034-025-00900-w

**Published:** 2025-05-09

**Authors:** Tecelli Domínguez, Lourdes Nieto, Ana Fresán, Tamara Sheinbaum, Rebeca Robles

**Affiliations:** 1 Centro de Investigación en Salud Mental Global, Instituto Nacional de Psiquiatría “Ramón de la Fuente Muñiz”-UNAM, Mexico City, Mexico; 2 Laboratorio de Epidemiología Clínica, Subdirección de Investigaciones Clínicas, Instituto Nacional de Psiquiatría “Ramón de la Fuente Muñiz”, Mexico City, Mexico; 3 Dirección de Investigaciones Epidemiológicas y Psicosociales, Instituto Nacional de Psiquiatría “Ramón de la Fuente Muñiz”, Calzada México Xochimilco # 101, Col. San Lorenzo Huipulco, Tlalpan., 14370 Mexico City, Mexico

**Keywords:** Bullying, Self-esteem, Early psychosis, Psychosis risk symptoms, Ultra high-risk for psychosis, Psychosis continuum

## Abstract

**Background:**

Bullying has become a significant global health problem due to its high prevalence worldwide and long-term consequences on mental health, including the onset of psychotic symptoms. This study focuses on exploring the prevalence of bullying across three groups of Mexican individuals with different levels of psychosis risk symptoms: non-psychosis risk (non-PR), psychotic-like experiences (PLEs), and at clinically high-risk for psychosis (CHR-P). In addition, we compare sociodemographic features, self-esteem, and self-reported bullying between the groups and then examine whether these variables are associated with the probability of belonging to the PLEs or CHR-P groups compared to the non-PR.

**Methods:**

A general population sample completed the Prodromal Questionnaire-Brief (PQ-B) to determine the presence of PLEs. Those meeting the PQ-B cut-off threshold were assigned to the PLEs group (*n* = 490), while those who scored below the cut-off comprised the non-PR group (*n* = 1,125). The CHR-P group (*n* = 45) was an independent clinical sample meeting the criteria established by the Comprehensive Assessment of At-Risk Mental States. All participants completed self-reports of sociodemographic characteristics, bullying, and self-esteem.

**Results:**

The CHR-P group had a higher percentage of men, single participants, and lower levels of education than the PLEs and the non-PR groups. PLEs and CHR-P participants reported a lower socioeconomic status, lower self-esteem, and higher prevalence of bullying than the non-PR group. The multinomial logistic regression indicated that the factors associated with belonging to the CHR-P group were lower education, being a man, and being single. Furthermore, being younger, having lower self-esteem, and having experienced bullying were associated with belonging to the PLEs and CHR-P groups. Among all these variables, bullying emerged as a robust risk factor associated with psychosis risk symptoms since it increased the odds of being CHR-P by threefold compared to the non-PR group, and it also increased the risk of PLEs compared to the non-PR group.

**Conclusions:**

Findings highlight the relevance of prioritizing anti-bullying school-based programs to provide a safer school environment, as well as strengthening self-esteem (potential protective factor) in vulnerable individuals to reduce the risk of developing psychosis and minimize the long-term impact of bullying victimization on further mental health conditions.

## Background

Psychosis is characterized by an altered perception of reality, with individuals experiencing symptoms that may include hallucinations, false beliefs, disordered thoughts and speech, and a marked impairment in psychosocial functioning [1]. Before the onset of clinical or frank psychotic symptoms, there is a period of changes lasting from weeks to several years, referred to as a prodrome, in which attenuated or subthreshold psychotic features start to manifest [1–3]. The psychotic disorder prodrome has recently become the focus of prevention and early psychosis intervention programs worldwide, intended to identify people at clinically high risk of developing psychosis to improve the prognosis, slow its progression, ameliorate functional deficits, minimize the adverse effects of long-term antipsychotic medication, and improve the general well-being of patients [4–6].

Subthreshold psychotic symptoms are not exclusive to the prodrome or clinical high-risk stage of psychosis (CHR-P). They can occur in a variety of mental disorders [7] and are also common in the general population, with systematic reviews suggesting around 17% in children, 7–8% in adolescents, and 5–7% in adults [8]. Accordingly, the psychosis phenotype is expressed across a dynamic continuum of nonclinical (psychotic-like experiences), subclinical (clinical-high-risk), and clinical (psychotic disorders) manifestations, with varying degrees of dysfunction [9–11].

The risk of developing psychosis has been found to increase with exposure to several environmental factors, including traumatic events and/or stressful experiences during the lifespan [12]. Evidence in the literature indicates that childhood maltreatment and early trauma experiences represent robust and consistent predictors of psychotic symptoms during adolescence or adulthood across the psychosis continuum, from nonclinical expressions to psychotic disorders [13–15].

Bullying is among the most common types of violence experienced in childhood and adolescence and has become a significant global health problem due to its high prevalence of around 30 to 50% worldwide [16–19]. Childhood and adolescence are developmental phases in which identity and personality are being formed. Accordingly, bullying is associated with significant suffering and long-term consequences in the lives of victims, including educational problems (e.g., increased absenteeism, school failure, and school dropout) [20], lower self-esteem [21, 22], and the development of a wide variety of mental health conditions (e.g., depression, anxiety, post-traumatic-stress, self-harming behavior, suicidal ideation/behavior, psychotic symptoms, substance use disorders, sexual risk behavior, violent and delinquent behavior) [23–29].

Although the relationship between bullying and the emergence of early psychotic symptoms had not received much attention until relatively recently [13, 30], it has been suggested that bullying and peer victimization are socio-environmental factors associated with the onset of psychotic manifestations [31]. Studies in nonclinical and general population samples have indicated that being bullied during childhood increases the risk of developing psychotic experiences and/or psychotic symptoms in adolescence or later in adulthood and that the risk is greater when the frequency, severity, and persistence of bullying are increased [13, 17, 31–33].

The association between bullying experiences and psychotic symptoms has also been consistent across the psychosis continuum. Some studies have shown that, as compared with control groups, individuals experiencing a first psychotic episode were about twice as likely to report being bullied [34]. In CHR-P individuals, the prevalence of bullying was significantly higher (2–3 times) than in controls [17, 35–37]. Besides, within CHR-P individuals, those who experienced bullying showed a higher prevalence of psychiatric symptoms, including psychotic symptoms, and poorer premorbid and social functioning than those without bullying experiences [17, 36, 37].

While school bullying is considered a universal phenomenon, its prevalence and relation to psychotic disorders vary across cultures [32]. Mexico ranked first in school bullying in basic education internationally (with more than 18 million Mexican primary and lower secondary school students suffering from school violence) [38], however, studies focused on exploring the relationship between bullying and the onset or development of psychotic disorders in the Mexican population are very scarce. In fact, no recent national official data are available on the prevalence of psychotic disorders in Mexico, which along with the lack of early detection and intervention in psychosis programs, reveals that research and public policies addressed to psychotic spectrum disorders have not been a national priority.

Previous studies have found on the one hand, a high prevalence of CHR-P individuals (17.3%) in a Mexican general population sample [39] and, on the other hand, that bullying was a strong predictor of CHR-P [39, 40]. Building upon these earlier reports and considering that the high rates of bullying in Mexico may increase the risk of developing psychotic disorders, it is crucial to expand knowledge about the association of being bullied on the risk for psychotic manifestations across the psychosis continuum, in order to design and promote preventive intervention programs adapted to the sociocultural context of the Mexican population. Therefore, the present study aims to explore the prevalence of bullying across three groups of Mexican individuals with different levels of psychosis risk: non-psychosis risk (non-PR) group, psychotic-like experiences (PLEs) group, and at clinically high risk for psychosis (CHR-P) group. Focusing on different levels of risk within the same study may provide relevant insights to our understanding of the subclinical end of the psychosis continuum. In addition, this study aimed to extend previous research by investigating the association of self-esteem—a psychological factor associated with bullying and psychosis vulnerability in the international literature [21, 41]. Specifically, the present study first compares sociodemographic features, self-esteem, and self-reported bullying between the groups and then examines whether these variables are associated with the probability of belonging to the PLEs or CHR-P groups compared to the non-PR group (PLEs vs. non-PR; CHR-P vs. non-PR).

## Material and methods

### Design

This cross-sectional, exploratory, and comparative study of three groups: non-PR, PLEs, and CHR-P.

### Participants

The non-PR group comprised 1125 individuals from the general population with a mean age of 30.1 years (S.D = 8.8; range 15–52). The PLEs group included 490 individuals from the general population with a mean age of 27.3 years (S.D = 8.4; range 15–52) who endorsed > 6 PLEs or positive attenuated symptoms along with a distress score of ≥ 29 according to the cut-off used for the Spanish version of the Prodromal Questionnaire-Brief (PQ-B) [42]. Participants were excluded if they self-reported a psychotic disorder or a psychosis-related hospitalization. The CHR-P group comprised 45 individuals with a mean age of 22.6 years (S.D = 6.1; range 13–43) who met the CHR-P criteria established by the Comprehensive Assessment of At-Risk Mental States (CAARMS) [43]. At the time of the study, CHR-P participants were receiving psychological and/or psychiatric treatment.

### Procedure

Participants from the non-PR and PLEs groups completed an online survey through Qualtrics^®^ and provided informed consent before the assessment (minors provided informed assent and were authorized by their parents/guardians to participate). Recruitment was performed from March 2022 to October 2023. Participants from the CHR-P group were recruited through the Schizophrenia Clinic of the Ramón de la Fuente Muñiz National Institute of Psychiatry in Mexico City. Informed consent was obtained from all participants and minors’ parents or guardians. They were recruited from June 2019 to November 2022 and assessed through an interview by trained clinical psychologists. Study procedures were approved by the Research Ethics Committee of the Ramón de la Fuente Muñiz National Institute of Psychiatry (CEI/C/019/2021 and CEI-010-20170316) and adhere to the Declaration of Helsinki. None of the participants received financial compensation for their participation.

### Measures

Sociodemographic data include age, sex, marital/relationship status, educational level, occupation, and socio-economic status (based on monthly family income: low < 9,000 MXN; medium > 9,000 and < 45,000 MXN; high > 45,000 MXN [1 MXN is equivalent to approximately 0.05 USD]).

Psychotic-like experiences were assessed in the non-PR and PLEs groups with the PQ-B [42], a well-used self-reported scale of 21 items answered in a *yes/no* response format. All items answered affirmatively are further rated on a 5-point Likert distress scale.

CHR-P criteria were assessed only for the CHR-P group using the CAARMS [43], a semi-structured clinical interview designed to identify individuals at imminent risk for psychosis.

The self-report of being bullied was assessed by asking participants whether they had experienced bullying (dichotomous response option yes/no), adapted from the Questionnaire of Stressful Life Events (QSLE) [44].

Self-esteem was assessed in the three groups using the Rosenberg Self-Esteem Scale (RSES) [45]. This 10-item self-report questionnaire evaluates global self-esteem with questions about general feelings regarding the self. Five items are written positively, while the other five are negatively worded and reversely scored. RSES items are rated on a four-point Likert response scale (1 = strongly disagree to 4 = strongly agree). The scale ranges from 10 to 40.

### Statistical analyses

Descriptive information was reported by frequencies and percentages for categorical variables and means and standard deviations (S.D.) for continuous variables. First, all variables were compared between the non-PR, the PLEs, and the CHR-P groups using Chi-square tests (χ^2^) and analysis of variance (ANOVA) with Bonferroni correction when appropriate. The effect size was determined with Cramer’s *V* for categorical variables comparisons and the partial eta squared (ƞp^2^) for continuous variables comparisons, with reference values for the effect size estimation interpretation: 0.01 = small, 0.06 = medium, 0.14 = large [46].

After the comparative analyses, a multinomial logistic regression was performed using the groups as dependent variables, with the non-PR group used as a reference value. Demographic variables, self-report of bullying, and self-esteem were included as independent variables. The Pearson chi-square test was used to determine the model’s goodness of fit, and the Nagelkerke R2 was determined to identify the variance explained by the model. Regression coefficients (β), standard deviations of β, odds ratios, and 95% confidence intervals are reported. All analyses were performed using the SPSS version 21 for Windows, PC, and the alpha value for tests was set at p *≤* 0.05.

## Results

### Demographic features

As shown in Table 1, a higher percentage of men and single participants were found in the CHR-P group compared to the non-PR and the PLEs groups (p *≤* 0.001), comprised of more women and partnered participants. No differences arose between the non-PR and the PLEs groups in these variables (p *≥* 0.05). The CHR-P group has the youngest participants, followed by those in the PLEs and the non-PR groups, with the highest mean age of the three groups (*p* < 0.001, Bonferroni corrections *≤* 0.002). A higher number of PLEs and CHR-P participants reported a low socioeconomic status than the non-PR group. A significantly higher number of non-PR participants completed at least high school, followed by those in the PLEs group (95.2% and 90.0%, respectively) and the CHR-P group (73.3%).

Bullying was reported by 55.2% (*n* = 916) of all participants, with a higher prevalence in those from the PLEs and the CHR-P groups (65.1% and 71.1%, respectively). Participants from the non-PR group reported bullying the least frequently (50.2%, *p* < 0.001). Self-esteem was higher in the non-PR group participants (F = 155.2, *p* < 0.001) and lower in those in the PLEs and the CHR-P groups (with similar scores in these groups).

**Table 1:** Comparison of demographic features, self-reported bullying, and self-esteem between groups

	Non-PR *n* = 1125	PLEs *n* = 490	CHR-*P* *n* = 45	Statistic
*Sex n (%)*				
Men	249 (22.1)	96 (19.6)	22 (48.9)	χ2 = 20.5, df 2, *p* < 0.001
Women	876 (77.9)	394 (80.4)	23 (51.1)	Cramer’s *V* = 0.11
*Age (years) Mean (S.D.)*	30.1 (8.8)	27.3 (8.4)	22.6 (6.1)	F_(2)_ = 31.3, df 1654, *p* < 0.001 ƞp^2^ = 0.04
*Marital status n (%)*				
Single	642 (57.1)	310 (63.3)	42 (93.3)	χ2 = 27.0, df 2, *p* < 0.001
Partnered	483 (42.9)	180 (36.7)	3 (6.7)	Cramer’s *V* = 0.12
*Socioeconomic status n (%)*				
Low	313 (27.8)	177 (36.1)	15 (33.3)	χ2 = 11.2, df 2, *p* = 0.04
Medium/medium-high	812 (72.2)	313 (63.69)	30 (66.7)	Cramer’s *V* = 0.08
*Level of education n (%)*				
Up to secondary school	54 (4.8)	49 (10.0)	12 (26.7)	χ2 = 42.2, df 2, *p* < 0.001
High school or more	1071 (95.2)	441 (90.0)	33 (73.3)	Cramer’s *V* = 0.16
*Occupation n (%)*				
Unemployed/none	63 (5.6)	35 (7.1)	3 (6.7)	
Students/employed	1062 (94.4)	455 (92.9)	42 (93.3)	χ2 = 1.4, df 2, *p* = 0.48
*Bullying-self-report n (%)*				
No	560 (49.8)	171 (34.9)	13 (28.9)	χ2 = 35.3, df 2, *p* < 0.001
Yes	565 (50.2)	319 (65.1)	32 (71.1)	Cramer’s *V* = 0.14
Rosenberg Self-esteem Scale *Mean (S.D.)*	29.2 (6.6)	23.3 (5.9)	23.2 (6.2)	F_(2)_ = 155.2, df 1654, *p* < 0.001 ƞp^2^ = 0.14

The multinomial logistic regression displays adequate goodness of fit (*p* > 0.05) and a significant contribution of the variables to the model (Nagelkerke R^2^ = 26.6; see Table 2). The model shows that the CHR-P are more likely to be men (OR = 2.45), less frequently partnered (OR = 0.17), and with a lower level of education (OR = 5.51) than individuals in the non-PR group. Also, both PLEs and CHR-P individuals are more likely to be younger (OR = 0.97 and OR = 0.93, respectively) and more frequently report having suffered from bullying (OR = 1.39 and OR = 3.32, respectively), and have lower self-esteem (OR = 0.878 and OR = 0.87 in both groups).

This means that each year less of age (1.03 and 1.07 times), each 1-point scoreless on the self-esteem assessment (1.14 and 1.13 times), and a history of bullying are associated with individuals with PLEs or CHR-P in contrast to those belonging to the non-PR group. Being a man, being single, and having a lower level of education increased the probability of being in the CHR-P group exclusively.

**Table 2:** Multinomial logistic regression analysis including demographic, self-reported bullying and self-esteem for identifying group belonging

	(β)	(β) S.D.	OR	95% C.I.	(β)	(β) S.D.	OR	95% C.I.
	PLE’s vs. non-PR				CHR-*P* vs. non-PR			
Sex—man	− 0.17	0.15	0.84	0.62–1.13	0.89*	0.32	2.45	1.29–4.64
Age (years)	− 0.02*	0.008	0.97	0.96–0.99	− 0.06*	0.02	0.93	0.88–0.98
Marital status—single	− 0.07	0.12	1.39	1.08–1.79	− 1.75*	0.61	0.17	0.05–0.57
Socioeconomic status—low	− 0.07	0.12	0.93	0.72–1.19	0.19	0.34	1.20	0.61–2.36
Level of education *≤* secondary	0.28	0.31	1.33	0.71–2.47	1.70*	0.62	5.51	1.62–18.65
Occupation—unemployed/none	− 0.05	0.24	0.94	0.59–1.52	− 0.08	0.64	0.92	0.26–3.25
Bullying—Yes	0.33*	0.12	1.39	1.08–1.79	1.20*	0.47	3.32	1.32–8.34
Self-esteem (score)	− 0.13**	0.01	0.87	0.86–0.89	− 0.13**	0.02	0.87	0.82–0.92

*p ≤ 0.01; **p ≤ 0.001

Pearson Chi square *p* = 0.96; Nagelkerke R^2^ = 0.26

## Discussion

This study aimed to analyze differences between Mexicans presenting nonclinical and subclinical manifestations of psychotic risk symptoms and explore the associations of bullying, self-esteem and other sociodemographic risk/protective factors across different levels of psychosis proneness, which can contribute to providing evidence-based information for targeting preventive intervention programs adapted to the sociocultural context and needs of Mexican individuals vulnerable to psychosis who were also victims of bullying.

Findings showed that the CHR-P group was mainly comprised of younger single men with lower levels of education than the PLEs and the non-PR groups. Moreover, PLEs and CHR-P participants reported lower socioeconomic status, lower self-esteem, and a higher prevalence of bullying than the non-PR group. This is consistent with evidence from early psychosis research, which indicates that male gender, younger age, lower education, poor socioeconomic status, lower self-esteem, and having been bullied were important factors associated with vulnerability to psychosis [5, 13, 17, 35, 41, 47–50].

The factors associated with belonging to the CHR-P group were lower education, being a man, and being single. Furthermore, being younger, having lower self-esteem, and having experienced bullying were associated with belonging to the PLEs and CHR-P groups. Among all these variables, bullying emerged as a robust risk factor associated with psychosis risk since it increased three times the probability of being CHR-P compared to the non-PR group, and it also increased the risk of PLEs compared to the non-PR group. This supports previous studies suggesting that bullying may be involved in the development of psychotic phenomena as a stressful risk factor or represent a developmental marker of risk for later psychotic experiences and CHR-P symptoms [17, 31, 33, 50, 51]. Further research in the Mexican population examining the dose effect of frequency and duration of different bullying subtypes on early psychotic symptoms would be of particular interest and importance [32]. Taking into account the potential bi-directionality of the association between bullying and psychotic symptoms, it is also important to explore if those experiencing psychotic risk symptoms are more vulnerable to bullying victimization, as has been suggested in previous studies [30, 36, 50].

Considering the association of bullying with the risk of subthreshold psychotic symptoms, along with the high prevalence of bullying reported overall (55.2%; *n* = 916), and especially by those from the PLEs (65.1%) and the CHR-P groups (71.1%), it is crucial to promote and prioritize preventive strategies to reduce the rates of peer bullying victimization in Mexican schools. It is recommended that both teachers and pupils be aware of the long-term effects of bullying on mental health in general and in those at high risk of psychosis in particular [30, 32].

Findings also provide evidence that supports previous studies about the important role of low self-esteem as a risk factor for increased susceptibility to psychotic symptoms [41]. It seems that self-esteem could become a protective factor if early psychosis interventions are focused on strengthening and increasing it. Moreover, since low self-esteem is closely related to being a victim of bullying [25], preventive bullying strategies could address the reinforcement of self-esteem in children and adolescents [21, 22], which, in turn, might also be protective against the onset of psychotic risk symptoms later in life.

### Limitations

Some of the limitations of the present study are related to the cross-sectional design and the use of self-reported questionnaires, specifically for assessing bullying with a single binary item. It would have been desirable to examine the effect of frequency and duration of different bullying subtypes to enrich the information obtained. However, for the specific purpose of this study, the use of binary question was useful as an initial exploratory assessment of the prevalence of bullying and made it possible to use the odds ratio for a more meaningful, interpretable, and realistic measure of strength of its association with psychosis risk symptoms [52]. Another limitation was the use of self-reported questionnaire for assessing PLEs (PQ-B) and the likely overlap between the PLEs and CHR-P groups, due to the meaningful proportion of the PLEs participants who might meet CHR-P criteria. We attempted to mitigate this overlap by using two different measures for each group. The CHR-P group included psychiatric help-seeking individuals assessed with the CAARMS, while the PQ-B was used for screening purposes to assess the severity of PLEs and identify from a general population those who could be at higher risk of developing psychosis and those who did not (based on the cut-off established for severity of symptoms and distress associated). However, we are not able to determine with the PQ-B if the PLEs participants meet the CHR-P criteria, because other important psychosis risk indicators need to be considered, such as functional impairment, family history of psychosis and/or schizotypal personality disorder (which are considered in the CAARMS criteria). Therefore, findings should be interpreted with caution (e.g., for group comparisons or generalizability).

In addition, considering the age differences between the groups, it is important to take into account the possible biases of the retrospective assessment of bullying, such as the influence of memory loss, causal inferences, distortion and subsequent life experiences, as well as the possible false positive or negative classification due to the potential effect of the presence of mental health problems in adulthood and the reminiscence of early bullying experiences [53]. To better understand the mechanism underlying the relationship between bullying and psychosis risk, it is recommended for further studies to explore longitudinally the developmental trajectories involved in this relationship and the interaction between personal, social, and biological factors [31].

### Clinical implications and recommendations for preventive strategies

In line with prior research, the findings of the present study highlight the relevance of developing school-based intervention programs designed for bullied students to achieve early prevention of psychotic disorders [32], as well as strengthen self-esteem, promote resilience-building, and provide a safer, healthy, and a more inclusive school environment for pupils with the hope of reducing the risk of developing psychosis in vulnerable individuals and minimizing the long-term impact of bullying victimization on further mental health conditions [16, 33, 54].

Given that children and adolescents spend most of the day and a considerable portion of their lives in school settings, educational institutions have a key role in preventing bullying [20, 55]. Those working as school psychologists and school counselors can play an important role in directing students and families toward effective and specialized interventions [56]. Evidence suggests positive benefits of school anti-bullying programs in high-income countries [20]. However, more implementation research in low- and middle-income countries is needed to confirm whether existing programs may be successful in different educational, cultural, and socioeconomic contexts [57].

Based on a preventive perspective, it is important to improve the early detection of psychotic experiences through community, primary care, and school programs in children and adolescents victims of bullying [31]. In addition, clinicians have an essential role in monitoring and performing routine screening to detect previous experiences of traumatic events, such as maltreatment and bullying in those reporting early symptoms of psychosis, to provide adequate treatment that minimizes harm to victims and reduces the associated risk for psychiatric disorders [33, 54]. Interventions and specialized support to the victims can be intended to lessen their psychological distress, improve self-esteem and emotion regulation processes, enhance social skills and increase their wellbeing [29]. Studies have recently focused on Cognitive Bias Modification Therapy, a novel psychological intervention with promising preliminary results in patients with psychotic symptoms [58], that could be adapted to fit the school context and target bullying victims at risk for psychosis to enable them to cope with their bullying experiences and reduce its long-term impact [32].

Finally, to reduce and prevent bullying in schools, it is necessary to raise awareness of the adverse consequences of violence on the mental health of children and adolescents that persist and have repercussions during the lifespan, promote collaborative work between pupils, families, teachers, school authorities, and mental health professionals, as well as the integration of anti-bullying programs into education, health, and development priorities to prevent both perpetration and bullying victimization [16, 29, 50, 55, 57]. To improve the reach of bullying prevention strategies, public policies may include the development of educational, attractive, and teen-friendly videos against bullying to be disseminated through public media and social networks since their impact on the population in general and, in particular, on youth has turned out to be very strong [59].

## Data Availability

The data sets presented in this article are not readily available due to ethical restrictions regarding the privacy of research participants. The data is available upon reasonable request to the corresponding author.
